# Predicting changes in protein thermostability brought about by single- or multi-site mutations

**DOI:** 10.1186/1471-2105-11-370

**Published:** 2010-07-02

**Authors:** Jian Tian, Ningfeng Wu, Xiaoyu Chu, Yunliu Fan

**Affiliations:** 1 Biotechnology Research Institute, Chinese Academy of Agricultural Sciences, Beijing 100081, China

## Abstract

**Background:**

An important aspect of protein design is the ability to predict changes in protein thermostability arising from single- or multi-site mutations. Protein thermostability is reflected in the change in free energy (ΔΔ*G*) of thermal denaturation.

**Results:**

We have developed predictive software, Prethermut, based on machine learning methods, to predict the effect of single- or multi-site mutations on protein thermostability. The input vector of Prethermut is based on known structural changes and empirical measurements of changes in potential energy due to protein mutations. Using a 10-fold cross validation test on the M-dataset, consisting of 3366 mutants proteins from ProTherm, the classification accuracy of random forests and the regression accuracy of random forest regression were slightly better than support vector machines and support vector regression, whereas the overall accuracy of classification and the Pearson correlation coefficient of regression were 79.2% and 0.72, respectively. Prethermut performs better on proteins containing multi-site mutations than those with single mutations.

**Conclusions:**

The performance of Prethermut indicates that it is a useful tool for predicting changes in protein thermostability brought about by single- or multi-site mutations and will be valuable in the rational design of proteins.

## Background

Improving protein thermostability is an important goal of protein engineering [1]; by making enzymes easier to handle, increased thermostability can increase storage options and expand the temperature range of applications, and facilitate the commercial development of enzymatic products [2-5]. Mutations at certain residues can significantly alter a protein's structure and thermostability [6,7]. The free energy (Δ*G*) of denaturation can be altered by single- or multi-site mutations; the change in Δ*G *(ΔΔ*G*), an indication of the change in protein thermostability, has been determined for many mutated proteins by the thermal denaturation method [1]. These data have been collected and deposited in publicly available databases [6,8,9]. From these data it is possible to develop computational methods to identify mutations *in silico *that could improve protein thermostability.

Various methods [2,10] have been proposed to predict thermostability changes brought about by protein mutations; these methods have been based on changes in structural energy [4,11,12], statistical analyses of mutant protein thermostability [13,14], and machine learning [15-21]. Methods based on changes in structural energy typically attempt to analyze changes in physical energy potentials [15], either by calculation, statistical analysis, or empirical measurement, with the objective of understanding the effects of mutations by comparing the energy difference between the wild-type and mutant structures [22,23].

Recently, various machine learning approaches based on support vector machines (SVM) [18,19,24], neural networks [21], and decision trees [16] have been proposed for predicting the effects of mutations on thermostability [8,9]. These approaches typically use large datasets of known primary, secondary, and tertiary structures of proteins to train the complex nonlinear functions.

Most approaches to predicting stability changes caused by mutations focus on a small number of mutations in a protein, often at a single site [2,23]. However, many factors, such as hydrophobicity, van der Waals interactions, hydrogen bonds, ion pairs, and non-covalent interactions, contribute to protein thermostability [25]. Thus, multi-site mutations would typically be expected to have a greater and more complex effect on protein thermostability than can be determined from single-site mutations alone [26,27].

It is thus necessary to have a reliable method for discriminating between stabilizing and destabilizing mutations, as well as for predicting the effects of single- and multi-site mutations on the thermostability of proteins. In this study, we introduce the program "Prethermut" (Predicting changes in protein thermostability brought about by single- or multi-site mutations), which predicts protein thermostability changes caused by single- or multi-site mutations. The program uses machine learning to construct classification models (for predicting only the sign of ΔΔ*G*) and regression models (for predicting the actual value of ΔΔ*G*). The input feature of Prethermut was developed from structural energy calculations derived from empirical measurements of energy potentials and certain structural attributes reflecting non-covalent interactions between residues within the 3-D structure. Two large non-redundant datasets, the M-dataset and S-dataset, were used to train Prethermut and test its robustness, respectively.

## Results and Discussion

### Training and validation

To train the models of Prethermut, a dataset (M-dataset) was constructed, containing data from 3366 mutants. In the M-dataset, 836 mutants had increased stability, with a mean ΔΔ*G *of 1.50 ± 1.36 kcal/mol, and 2530 mutants had decreased stability, with a mean ΔΔ*G *of -1.77 ± 1.03 kcal/mol. The number of mutation sites in the M-dataset ranged from 1 to 9 (Table 1). The input features of Prethermut were calculated from the structural features listed in Table 2, which include structural energies calculated from empirical measurements of energy potentials [11,28] and certain structural attributes reflecting non-covalent interactions between residues in the 3-D structure [29].

**Table 1:** Classification and regression performance of Prethermut on the M-dataset

Method^a^	Mutation Numbers	n^b^	MCC	Q2 (%)	Sensitivity (%)	Specificity (%)	*r*
RF	1	2765	0.46	77.3	71.3	7 9.7	0.70
RF	2	441	0.66	84.8	81.0	86.5	0.79
RF	3	93	0.86	96.8	84.6	98.8	0.87
RF	≥4	67	0.92	97.0	93.8	98.0	0.86
RF	≥1	3366	0.50	79.7	73.6	81.1	0.72
SVM	1	2765	0.39	79.8	41.2	92.1	0.64
SVM	2	441	0.59	83.0	51.1	97.4	0.74
SVM	3	93	0.45	89.7	23.1	100.0	0.79
SVM	≥4	67	0.66	88.1	50.0	100.0	0.78
SVM	≥1	3366	0.43	79.7	42.7	93.2	0.67

All of the results were obtained by a 10-fold cross validation on the M-dataset. See Methods for definitions of overall accuracy (Q2), Matthews correlation coefficient (MCC), sensitivity, specificity, and Pearson correlation coefficient (*r*). ^a^The number of trees in the random forests (RF) method is 10000; the parameters for the support vector machine (SVM) method are gamma (*g*) = 2, cost (*c*) = 8, and the weight for the positive samples (*w*) = 3. ^b^n is the number of mutant proteins in the sample; the total number of proteins in the M-dataset was 3366.

**Table 2:** Structural features used in Prethermut

Feature	Program^a^	Feature	Program
Total energy	FoldX	Stereochemical improper dihedral potential	Modeller 9.7
Backbone H-bond	FoldX	Frequency_[0,2.1) ^b^	Modeller 9.7
Sidechain H-bond	FoldX	Frequency_[2.1,2.2)	Modeller 9.7
Van der Waals forces	FoldX	Frequency_[2.2,2.3)	Modeller 9.7
Electrostatic attractions	FoldX	Frequency_[2.3,2.4)	Modeller 9.7
Solvation polar	FoldX	Frequency_[2.4,2.5)	Modeller 9.7
Solvation hydrophobic	FoldX	Frequency_[2.5,2.6)	Modeller 9.7
Van der Waals clashes	FoldX	Frequency_[2.6,2.7)	Modeller 9.7
Entropy side chain	FoldX	Frequency_[2.7,2.8)	Modeller 9.7
Entropy main chain	FoldX	Frequency_[2.8,2.9)	Modeller 9.7
Torsional clash	FoldX	Frequency_[2.9,3.0)	Modeller 9.7
Backbone clash	FoldX	Frequency_[3.0,3.1)	Modeller 9.7
Helix dipole	FoldX	Frequency_[3.1,3.2)	Modeller 9.7
Current energy	Modeller 9.7	Frequency_[3.2,3.3)	Modeller 9.7
Bond energy	Modeller 9.7		

^a^The corresponding feature was calculated by the programs (FoldX [11,28] and Modeller 9.7 [29]). ^b^The frequency of short non-covalent contacts with a distance of less than 2.1 Å.

The classifiers of random forests (RF) and support vector machines (SVM) were trained on the M-dataset to predict whether the mutations were stabilizing or destabilizing (i.e., the sign of ΔΔ*G*). The regression methods of random forest regression (RFR) and support vector regression (SVR) were used to predict the change in free energy (ΔΔ*G*) of thermal denaturation of the mutant proteins. Because the number of mutants in the training set having increased thermostability was disproportionately small versus those with decreased thermostability (by a factor of approximately three), the down sampling approach [30] was used for RF implementation, and for SVM implementation the weight given to the mutants with increased thermostability was 3-fold greater than that given to the mutants with decreased thermostability. The performance of the methods was assessed by a 10-fold cross validation on the M-dataset (Table 1).

**Figure 1:**
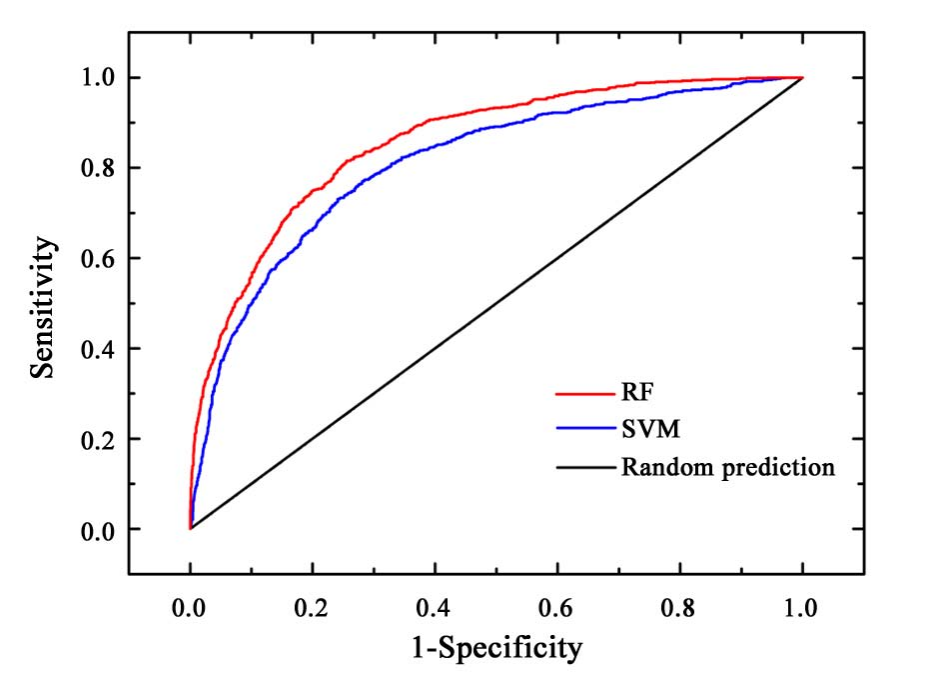
**Receiver operating characteristic curves for random prediction and the prediction of Prethermut using the random forests (RF) and support vector machines (SVM) methods**. The curves were obtained from the 10-fold cross validation test on the M-dataset.

The classifiers of RF and SVM yielded a similar overall accuracy (Q2) of 79.7% on the M-dataset. However, the Matthews correlation coefficient (MCC) of the RF classifier was 0.50, while that of the SVM classifier was 0.43. This indicates that the RF classifier was better at distinguishing between stabilizing and destabilizing mutations. The better performance of the RF classifier was probably due to the imbalance of the two classes in the M-dataset and may indicate that the RF algorithm was better at accommodating this imbalance than SVM. To further investigate the robustness of the SVM and RF classifiers, receiver operating characteristic curves were plotted based on 10-fold cross validation tests on the M-dataset (Figure 1). The values for the area under the curve for the SVM and RF classifiers were 0.86 and 0.81, respectively. These results indicate that the RF and SVM classifiers could be used to predict which mutations were stabilizing or destabilizing and that the RF classifier was a better performer than the SVM classifier.

The importance of each variable to the input vector of Prethermut was also assessed by evaluating the decrease in the classification accuracy of RF [30,31]. As shown in Additional file 1: Table S1, all structural features contributed to the predictor, with the most important feature being the total energy, as calculated by FoldX [11,28].

**Figure 2:**
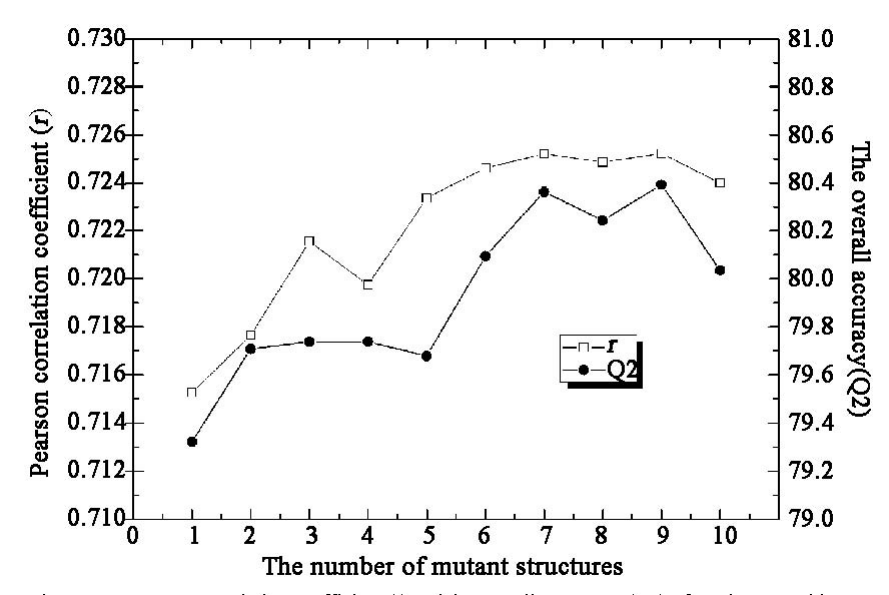
**Pearson correlation coefficient (*r*) and the overall accuracy (Q2) of Prethermut with different numbers of mutant structural features used in the input vector**. The results were calculated on the M-dataset with 10-fold cross validation by the random forests method.

As described in the Methods section, the input vector of Prethermut was calculated on the basis of *k *different structural features of a mutant protein. Here, we evaluated the effect of using different numbers of structural features to build the input vector. As shown in Figure 2, Q2 and the Pearson correlation coefficient (Pearson's *r*) for regression became balanced when the value of *k *was greater than 6. We also tested the effect of different numbers of classification trees in the RF. As shown in Additional file 1: Table S2, the performance of Prethermut was not affected when the number of trees was greater than 500.

**Figure 3:**
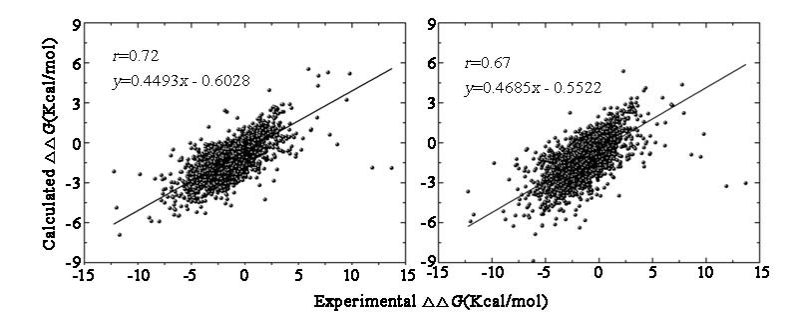
**Pearson correlation coefficient (*r*) and equation of the regression line (*y*) of Prethermut using the algorithms of support vector regression and random forest regression**. The results were calculated on the M-dataset with 10-fold cross validations by the random forests regression method (left panel) and support vector regression method (right panel).

Two regression predictors were trained to directly estimate the ΔΔ*G *values by the SVR and RFR algorithms. Regression performance was evaluated based on the results of 10-fold cross validation of the M-dataset (Figure 3). The SVR predictor was trained based on the Radial Basis Function (RBF) kernel with parameters gamma (*g*) = 2 and cost (*c*) = 8. Pearson's *r *of the SVR-predicted and experimental data was 0.67. The results of RFR (Table 1, Figure 2) showed better performance than SVR. Pearson's *r *of the RFR-predicted and experimental data was 0.72.

### Prediction accuracy using different numbers of mutation sites

We examined the performance of Prethermut in predicting the changes in thermal stability of mutant proteins containing different numbers of mutations. As shown in Table 1, the classification or regression accuracy was better with a larger number of mutations. For example, Q2 and Pearson's *r *for the prediction of thermostability of proteins containing three mutations, as predicted by RF or RFR, were 96.8% and 0.87, respectively, which was better than the results obtained with proteins having one or two mutations. We also calculated the average absolute value of ΔΔ*G *of the mutant proteins having different numbers of mutations. The average absolute value of ΔΔ*G *for proteins carrying one, two, three, or more than three mutations was 1.50 kcal/mol, 1.94 kcal/mol, 2.04 kcal/mol, and 2.28 kcal/mol, respectively. These results indicate that the change in protein thermostability was greater with increasing number of mutations. The prediction accuracy was also evaluated as a function of the magnitude of absolute ΔΔ*G*. As shown in Table 3, the larger the value of absolute ΔΔ*G*, the greater the accuracy of the prediction.

**Table 3:** Performance of Prethermut on the M-dataset with different ranges of absolute ΔΔ*G*

Method^a^	Range of absolute ΔΔ*G*	m^b^	MCC	Q2 (%)	Sensitivity (%)	Specificity (%)	*r*
RF	[0, 1)	1466	0.33	66.8	68.9	65.5	0.39
RF	[1, 2)	873	0.57	84.0	78.7	85.2	0.56
RF	[2, 3)	509	0.66	91.0	88.1	91.3	0.69
RF	[3, 14)	518	0.77	94.8	87.9	95.7	0.72
SVM	[0, 1)	1466	0.28	68.3	36.9	87.1	0.31
SVM	[1, 2)	873	0.52	86.3	49.7	95.0	0.55
SVM	[2, 3)	509	0.64	93.3	57.6	98.0	0.65
SVM	[3, 14)	1466	0.62	93.4	44.8	99.6	0.63

All results were obtained by a 10-fold cross validation on the M-dataset. See Methods for definitions of overall accuracy (Q2), Matthews correlation coefficient (MCC), sensitivity, specificity, and Pearson correlation coefficient (*r*). ^a^The number of trees in the random forests (RF) method is 10000; the parameters for the support vector machine (SVM) method are gamma (*g*) = 2, cost (*c*) = 8, and the weight for the positive samples (*w*) = 3. ^b^m is the number of mutant proteins in the M-dataset that have the same range of absolute ΔΔ*G*.

### Reliability index of classification by Prethermut

**Figure 4:**
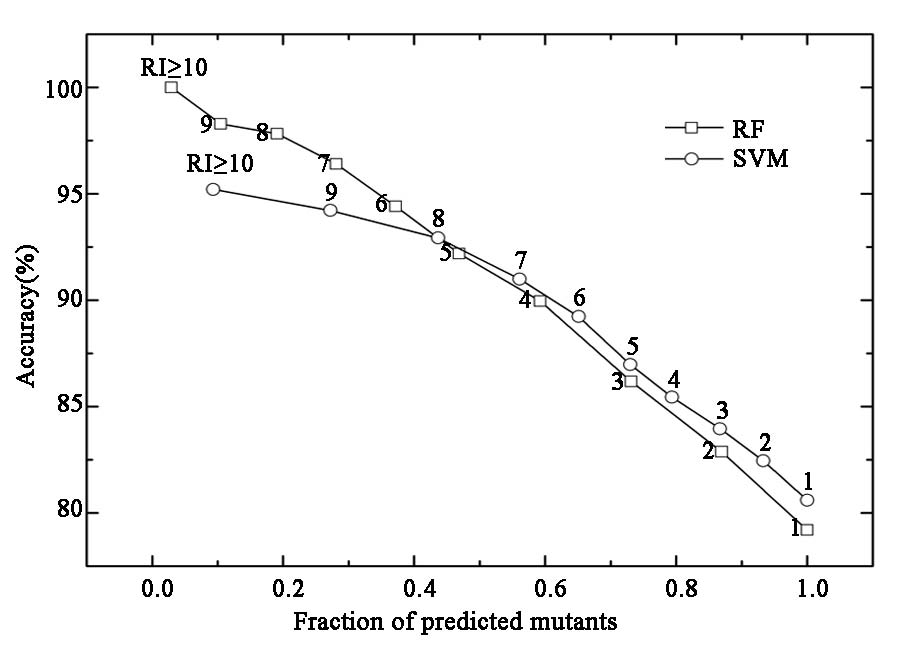
**Average prediction accuracy calculated cumulatively with a reliability index (RI) above a given value**. The results were based on the M-dataset with 10-fold cross validation by the random forests (RF, squares) method and support vector machine (SVM, circles) method.

When machine learning is used to classify samples, it is important to know the reliability of the prediction results [24,32,33]. In this study, a reliability index (RI) was assigned to a prediction, depending on the output of SVM or RF. The output *O *of SVM or RF ranged from zero to one, and the RI value was computed as RI = INTEGER(20 × abs(*O*-0.5)). Thus, the RI value reflects, on a scale of zero to ten, the degree of certainty of the classification; as the output *O *approaches the extreme of zero or one, the RI value approaches its maximum value. Figure 4 shows the expected prediction accuracies and the fraction of mutants yielding a given RI value. For example, approximately 28% of the mutants had an RI ≥ 7 for the RF method and of these, 96% were correctly predicted. All of the results were obtained by SVM or RF with 10-fold cross validation of the M-dataset.

### Comparison with other methods

Professor Gideon Schreiber [2] constructed an independent dataset and systematically assessed the performance of the frequently used computational methods of CC/PBSA [4], EGAD [12], FoldX [11], Hunter [32], I-Mutant2.0 [20], Rosetta [34] and the Combining method [2]. We chose the Schreiber dataset (S-dataset) to test the performance of Prethermut to compare it with the published results from these other methods. As shown in Table 4, Prethermut predicted the thermostability of all the mutant proteins in the S-dataset having known wild-type structure with a better classification and regression accuracy than any of the other methods. This excellent performance of Prethermut was due to its efficient machine learning methods and the more determinant structural features used as inputs.

**Table 4:** Performance of Prethermut and other computational methods on the S-dataset

Method	*r*	Q2 (%)	n^a^
CC/PBSA	0.56	78.6	478
EGAD	0.59	71.0	1065
FoldX	0.5	69.5	1200
Hunter	0.45	69.4	1594
I-Mutant2.0	0.54	77.5	933
Rosetta	0.26	73.4	1913
Combining method	0.64	80.8	407
Prethermut (RF)^b^	0.72	78.6	2156
Prethermut (SVM)^c^	0.70	83.2	2156

See Methods for definitions of overall accuracy (Q2) and Pearson correlation coefficient (*r*). The prediction results of CC/PBSA, EGAD, FoldX, Hunter, I-Mutant 2.0, Rosetta, and Combining method were obtained from Potapov *et al. *[2]. ^a^n is the number of mutant proteins for which the method correctly predicted the change in thermostability. ^b^The number of trees in the Random forests (RF) method is 10000. The results were obtained by a 10-fold cross validation on the S-dataset. ^c^The parameters for the support vector machine (SVM) method are gamma (*g*) = 2, cost (*c*) = 4, and the weight for the positive samples (*w*) = 5. The results were obtained by a 10-fold cross validation on the S-dataset.

## Conclusions

Several predictors [2,16-18] have been constructed to predict the effect of a single mutation on protein thermostability, based on structural or sequence features. However, multi-site mutations usually have a greater effect on protein thermostability than single-site mutations [3]. In this study, we present a predictive computer program, called Prethermut, based on machine learning methods, that can directly predict the effect of single- and multi-site mutations on protein thermostability from the wild-type protein's structural features.

The high predictive power of Prethermut, assessed by a rigorous 10-fold cross validation procedure, is illustrated by a Q2 value of 79.7% for the classification of stabilizing and destabilizing mutations from the M-dataset, and a Pearson's *r *of 0.79 for the correlation between predicted and experimentally determined ΔΔ*G *values. The performance of Prethermut was also assessed in the independent S-dataset of more than 2000 mutants. Prethermut outperformed several published structure- and sequence-based predictors using the S-dataset. Although direct comparison of Prethermut with the other published predictors is not appropriate, because of differences in datasets used for training and testing, as well as the information used to develop the models, the results indicate that Prethermut is a powerful tool for predicting the effect of mutations on protein thermostability.

## Methods

### Datasets

In this study, two datasets (the M- and S-datasets) were used to train and test the validity of Prethermut. The first dataset, the M-dataset, consisting of the changes in free energy (ΔΔ*G*) of thermal denaturation of mutant proteins, was extracted from the ProTherm database [8,9] using three criteria:

(1) Both single- and multi-site mutations were considered.

(2) The protein structure was known at atomic resolution and had been deposited in the Protein Data Bank.

(3) Redundant data were removed, and an average free energy change (ΔΔ*G*) of the mutant was calculated when multiple data for the mutant, using the same experimental procedure, were available.

The final non-redundant M-dataset consisted of 3366 mutants with single- or multi-site mutations acquired from 129 different proteins. The ΔΔ*G *ranged from -12.23 kcal/mol to 13.7 kcal/mol. This dataset is available at http://www.mobioinfor.cn/prethermut/download.htm.

The second dataset, the S-dataset, was compiled by Dr. Vladimir Potapov [2] and obtained from http://ligin.weizmann.ac.il/~lpotapov/PEDS_mutants/mutants.html. This large, non-redundant dataset contained 2156 single-site mutants from 84 different proteins.

### Input vectors and encoding schemes

The essential step in applying machine learning methods to predict the sign or the actual value of ΔΔ*G *is to translate structural information into vectors with the fixed length, namely the encoding process. The input vectors for Prethermut were calculated as follows:

(1) For each mutant represented in the dataset, a wild-type structure was downloaded from the Protein Data Bank. All water molecules in the structures were manually removed.

(2) Structures of the mutants were modelled by the program Modeller 9.7, which uses the standard steps for building mutants [29]. It was supposed that *k *different structures of a mutant were modelled by Modeller 9.7 [29], as the Modeller program generates different mutant structures based on different random seeds [29].

(3) For training the model, the input vector of Prethermut contained 58 elements that were calculated from 29 structural features (Table 2), including the potential energies and physical characteristics of the features. In this study, the programs FoldX 3.0 [11] and Modeller 9.7 were used to calculate the values for these features, because these two programs have been widely used to predict and assess protein structures and are freely available.

(4) For each mutant, *k *different structures were modeled. The input vector ***M***_***i ***_of the *i*^th ^structure of the mutant was then calculated by FoldX 3.0 and Modeller 9.7, ***M***_***i ***_= [], where the value of *i *is from 1 to *k*. Then, all of the vectors ***M ***were averaged to yield a new vector ***N***, ***N ***= [*g*^*1*^, *g*^*2*^...*g*^*29*^].

(5) All of the residues in the wild-type protein were mutated via single site saturation mutagenesis by Modeller 9.7 [29]. It was supposed that the length of the wild-type protein is *l*, and then *l *× 19 mutants for the wild-type protein would be modeled by Modeller 9.7. The structural feature vector ***W***_***j ***_= [] for the *j*^th ^structure in all of the mutants of the wild-type protein was calculated, where the value of *j *is from 1 to l*19. The mean vector ***U ***= [*μ*^*1*^, *μ*^*2*^...*μ*^*29*^] and standard deviation vector ***S ***= [*σ*^*1*^, *σ*^*2*^...*σ*^*29*^] for all of the structural feature vectors ***W***_**j **_(*j *= 1, 2...l*20) were calculated.

(6) The final input vector ***Z ***= [*z*^*1*^, *z*^*2*^...*z*^*58*^] of Prethermut consisted of 58 elements and represented a combination of the two vectors (***V ***= [*v*^*1*^, *v*^*2*^...*v*^*29*^] and ***Y ***= [*y*^*1*^, *y*^*2*^...*y*^*29*^]). The element values (*v *and *y*) of vector ***V ***and ***Y ***were calculated as follows:(1)(2)

### Machine learning methods

In this study, the classification methods RF and SVM (for predicting the sign of ΔΔ*G*) and the regression methods RFR and SVR (for predicting the actual value of ΔΔG) were employed to train and test the robustness of the method, because these methods have been successfully used in many aspects of computational biology [20,35].

(1) RF. The RF is an ensemble machine learning methodology originated by Leo Breiman [30]. The basic idea of ensemble learning is to boost the performance of a number of weak learners via a voting scheme, where a weak learner can be an individual decision tree, a single perceptron/sigmoid function, or other simple and fast classifier [36]. Regarding RF, its hallmarks include bootstrap re-sampling, random feature selection, in-depth decision tree construction and out-of-bag error estimates [36].

(2) RFR. The RFR is built in a fashion similar to the classifier in RF [37], but the goal of RFR is to predict the continuous value of interest [38].

(3) SVM. SVM is used to identify the optimal hyperplane that separates two classes of samples [39,40]. SVM uses kernel functions to map the original data to a feature space of higher dimension and then locates an optimal separating hyperplane.

(4) SVR. In comparison with SVM, the objective of SVR is to estimate an unknown continuous valued function *y *= *f*(**X**), which is based on a finite number of samples [41,42]. The method has been successfully used in many bioinformatics tasks, such as predicting protein B-factors [42], residue contact numbers [43], and residue-wise contact orders [44].

The RF and RFR algorithms were run in the R programming environment (built by the R project, http://www.r-project.org/). To implement SVM and SVR, we used LIBSVM http://www.csie.ntu.edu.tw/~cjlin/ with an RBF kernel. The parameters of SVM or SVR were selected with the LIBSVM parameter selection tool http://www.csie.ntu.edu.tw/~cjlin/libsvmtools/.

### Prediction system assessment

Mutant proteins with a value of ΔΔ*G *> 0 for the thermal denaturation reaction were defined as positive samples, and the others were considered as negative samples. True positives (TP) and true negatives (TN) were identified as the positive and negative samples, respectively. False positives (FP) were negative samples identified as positive, and false negatives (FN) were positive samples identified as negative. The quality of the classification was determined based on sensitivity (TP/(TP+FN)), specificity (TN/(TN+FP)), overall accuracy (Q2), and the Matthews correlation coefficient (MCC). The Q2 and MCC were calculated as follows:(3)(4)

The regression quality for predicting the absolute value of ΔΔ*G *was evaluated by Pearson's *r*, calculated as follows:(5)

where *r *is Pearson's *r*, and *N*, *X*, and *Y *are the number of data, experimental ΔΔ*G *value, and predicted ΔΔ*G *value, respectively.

## Availability and requirements

Project name: Prethermut

Project home page: http://www.mobioinfor.cn/prethermut

Operating systems: Linux

Programming language: Perl

Required prerequisite programs: Perl version 5.6 or higher; Foldx 3.0; Modeller v9.7 or higher.

License: GNU General Public License. This license allows the source code to be redistributed and/or modified under the terms of the GNU General Public License, as published by the Free Software Foundation. The source code for the application is available at no charge.

Any restrictions to use by non-academics: None

## Authors' contributions

JT wrote the code of Prethermut. NW and YF supervised the work. JT, NW, and XC were involved in the preparation of the manuscript. All authors read and approved the manuscript.

## Supplementary Material

Additional file 1 **Table S1**. Selected structural features and the contribution of these features. **Table S2**. Prediction performance of Random Forests with different tree number. Click here for file
