# Supplementary material for: Predicting changes in protein thermostability brought about by single- or multi-site mutations
Source: BMC Bioinformatics. 2010 Jul 2;11:370. doi: 10.1186/1471-2105-11-370 (PMC2906492; doi:10.1186/1471-2105-11-370)
Supplement: Additional file 1 — Table S1. Selected structural features and the contribution of these features. Table S2. Prediction performance of Random Forests with different tree number. [file 1471-2105-11-370-S1.DOC]

**Table S1**. Selected structural features and the contribution of these features

| Feature | Program^a^ | Contribution^b^ | | |
| --- | --- | --- | --- | --- |
|  |  | Vector ***V*** | Vector ***Y*** | Average contribution |
| Total energy | FoldX | 1.00 | 0.64 | 0.82 |
| Backbone hbond | FoldX | 0.62 | 0.55 | 0.59 |
| Sidechain hbond | FoldX | 0.55 | 0.65 | 0.60 |
| Van der Waals | FoldX | 0.59 | 0.47 | 0.53 |
| Electrostatics | FoldX | 0.70 | 0.55 | 0.63 |
| Solvation polar | FoldX | 0.48 | 0.50 | 0.49 |
| Solvation hydrophobic | FoldX | 0.72 | 0.53 | 0.62 |
| Van der Waals clashes | FoldX | 0.66 | 0.52 | 0.59 |
| Entropy side chain | FoldX | 0.51 | 0.48 | 0.49 |
| Entropy main chain | FoldX | 0.59 | 0.26 | 0.43 |
| Torsional clash | FoldX | 0.63 | 0.00 | 0.31 |
| Backbone clash | FoldX | 0.70 | 0.00 | 0.35 |
| Helix dipole | FoldX | 0.55 | 0.34 | 0.45 |
| Current energy | Modeller 9.7 | 0.57 | 0.56 | 0.56 |
| Bond energy | Modeller 9.7 | 0.63 | 0.60 | 0.62 |
| Stereochemical improper dihedral potential | Modeller 9.7 | 0.54 | 0.63 | 0.59 |
| FREQUENCY_[0,2.1) | Modeller 9.7 | 0.55 | 0.47 | 0.51 |
| FREQUENCY_[2.1,2.2) | Modeller 9.7 | 0.58 | 0.53 | 0.55 |
| FREQUENCY_[2.2,2.3) | Modeller 9.7 | 0.55 | 0.53 | 0.54 |
| FREQUENCY_[2.3,2.4) | Modeller 9.7 | 0.44 | 0.55 | 0.49 |
| FREQUENCY_[2.4,2.5) | Modeller 9.7 | 0.54 | 0.53 | 0.53 |
| FREQUENCY_[2.5,2.6) | Modeller 9.7 | 0.45 | 0.48 | 0.47 |
| FREQUENCY_[2.6,2.7) | Modeller 9.7 | 0.58 | 0.52 | 0.55 |
| FREQUENCY_[2.7,2.8) | Modeller 9.7 | 0.54 | 0.48 | 0.51 |
| FREQUENCY_[2.8,2.9) | Modeller 9.7 | 0.56 | 0.54 | 0.55 |
| FREQUENCY_[2.9,3.0) | Modeller 9.7 | 0.41 | 0.40 | 0.41 |
| FREQUENCY_[3.0,3.1) | Modeller 9.7 | 0.56 | 0.38 | 0.47 |
| FREQUENCY_[3.1,3.2) | Modeller 9.7 | 0.51 | 0.43 | 0.47 |
| FREQUENCY_[3.2,3.3) | Modeller 9.7 | 0.40 | 0.34 | 0.37 |

^a^The corresponding feature was calculated by the program; ^b^The contribution of the features was calculated by the algorithm of Random forest with the 10000 tree and the values were standardized, which was from 0 to 1. The vector ***V*** and ***Y*** was calculated as shown in the part of Materials and Methods.

**Table S2.** Prediction performance of Random Forests with different tree number.

| Tree number | *r* | MCC | Q2 |
| --- | --- | --- | --- |
| 100 | 0.72 | 0.50 | 79.17 |
| 200 | 0.72 | 0.51 | 79.53 |
| 300 | 0.72 | 0.51 | 79.95 |
| 400 | 0.72 | 0.52 | 80.04 |
| 500 | 0.72 | 0.52 | 80.18 |
| 600 | 0.72 | 0.52 | 80.24 |
| 700 | 0.72 | 0.52 | 80.01 |
| 800 | 0.72 | 0.52 | 80.15 |
| 900 | 0.72 | 0.52 | 80.12 |
| 1000 | 0.72 | 0.52 | 79.92 |
| 2000 | 0.72 | 0.52 | 80.07 |
| 3000 | 0.72 | 0.52 | 80.12 |
| 4000 | 0.72 | 0.52 | 80.07 |
| 5000 | 0.72 | 0.52 | 80.07 |
| 6000 | 0.72 | 0.52 | 79.98 |
| 7000 | 0.72 | 0.52 | 80.04 |
| 8000 | 0.72 | 0.52 | 80.10 |
| 9000 | 0.72 | 0.52 | 80.10 |
| 10000 | 0.72 | 0.52 | 80.04 |

All of the results were obtained by a 10-fold cross validation on the M-dataset by the Random Forests (RF). For definitions of overall accuracy (Q2), Matthews correlation coefficient (MCC), Overall Accuracy (Q2), sensitivity, specificity and Pearson correlation coefficient (*r*), refer to the Methods section.
